# Navigating *in vitro* bioactivity data by investigating available resources using model compounds

**DOI:** 10.1038/s41597-019-0046-1

**Published:** 2019-04-29

**Authors:** Sten Ilmjärv, Fiona Augsburger, Jerven Tjalling Bolleman, Robin Liechti, Alan James Bridge, Jenny Sandström, Vincent Jaquet, Ioannis Xenarios, Karl-Heinz Krause

**Affiliations:** 1 Department of Pathology and Immunology, Medical School, University of Geneva, Geneva, Switzerland; 2 Vital-IT Group, SIB Swiss Institute of Bioinformatics, Lausanne, Switzerland; 3 Swiss-Prot group, SIB Swiss Institute of Bioinformatics, Medical School, Geneva, Switzerland; 4 SCAHT Swiss Centre for Applied Human Toxicology, Basel, Switzerland; 5 Center for Integrative Genomics, University of Lausanne, Lausanne, Switzerland; 6 Departement of Biochemistry and Chemistry, University of Geneva, Geneva, Switzerland

**Keywords:** Databases, Small molecules, Cheminformatics

## Abstract

The number of chemical compounds and associated experimental data in public databases is growing, but presently there is no simple way to access these data in a quick and synoptic manner. Instead, data are fragmented across different resources and interested parties need to invest invaluable time and effort to navigate these systems.

Both the CAS Registry^SM^ and PubChem^1^ contain more than 90 million compounds, with new compounds added daily. Most of these compounds are missing toxicological characterization, due in part to the limited capacity of current methods to assess a compound’s bioactivity in a living system. High-throughput and scalable *in vitro* test systems aim to bridge that gap. In combination with structural information and known molecular properties, these high-throughput data will allow researchers to describe toxicity pathways more comprehensively. However, the increasing amounts of new data presents its own set of challenges.

Anomalies in metadata records and the inadequate use of ontologies are hindering for the data to be FAIR^2^. Even after a compound has been published in a scientific document, the diversity of compound synonyms and identifiers, and lack of precise metadata and annotations, can lead to false conclusions and difficulties identifying the compound correctly^3^. To improve the reproducibility of experimental results and to test new hypotheses (e.g. development of predictive computational models), availability and accessibility of raw data are crucial. Using a set of four arbitrarily chosen model compounds (*aspirin*, *rosiglitazone*, *valproic acid*, and *tamoxifen*; Table 1), we investigated data access and consistency within publicly available online resources (Table 2). We observed that modest adoption of semantic web technologies and poor annotations of experimental metadata represent a major obstacle for high-quality data integration and reusability. We argue that this could be substantially improved by annotating compound-related experimental data with standardized ontologies. Also, new and existing resources should adapt to accommodate ontology-based data representation on their platforms and compounds should always be accompanied with a unique structural identifier that helps later discoverability and reduces mistakes.

**Table 1:** Table of model compounds used in the study and their identifiers including unified resource identifier (URI).

Aspirin	**ChEBI ID**	CHEBI:15365
	**PubChem CID**	CID2244
	**InChIKey**	BSYNRYMUTXBXSQ-UHFFFAOYSA-N
	**InChI**	InChI = 1S/C9H8O4/c1-6(10)13-8-5-3-2-4-7(8)9(11)12/h2-5H,1H3,(H,11,12)
	**SMILES**	CC(=O)Oc1ccccc1C(O)=O
	**ChEBI URI**	http://purl.obolibrary.org/obo/CHEBI_15365
Rosiglitazone	**ChEBI ID**	CHEBI:50122
	**PubChem CID**	CID77999
	**InChIKey**	YASAKCUCGLMORW-UHFFFAOYSA-N
	**InChI**	InChI = 1S/C18H19N3O3S/c1-21(16-4-2-3-9-19-16)10-11-24-14-7-5-13(6-8-14)12-15-17(22)20-18(23)25-15/h2-9,15H,10-12H2,1H3,(H,20,22,23)
	**SMILES**	CN(CCOc1ccc(CC2SC=O)NC2=O)cc1)c1ccccn1
	**ChEBI URI**	http://purl.obolibrary.org/obo/CHEBI_50122
Valproic acid	**ChEBI ID**	CHEBI:39867
	**PubChem CID**	CID3121
	**InChIKey**	NIJJYAXOARWZEE-UHFFFAOYSA-N
	**InChI**	InChI = 1S/C8H16O2/c1-3-5-7(6-4-2)8(9)10/h7H,3-6H2,1-2H3,(H,9,10)
	**SMILES**	CCCC(CCC)C(O)=O
	**ChEBI URI**	http://purl.obolibrary.org/obo/CHEBI_39867
Tamoxifen	**ChEBI ID**	CHEBI:41774
	**PubChem CID**	CID2733526
	**InChIKey**	NKANXQFJJICGDU-QPLCGJKRSA-N
	**InChI**	InChI = 1S/C26H29NO/c1-4-25(21-11-7-5-8-12-21)26(22-13-9-6-10-14-22)23-15-17-24(18-16-23)28-20-19-27(2)3/h5-18H,4,19-20H2,1-3H3/b26-25-
	**SMILES**	CC\C(c1ccccc1)=C(/c1ccccc1)c1ccc(OCCN(C)C)cc1
	**ChEBI URI**	http://purl.obolibrary.org/obo/CHEBI_41774

**Table 2:** A list of resources used in the study, their categorization and the number of estimated compounds in these resources at the time of the study.

Database	Number of compounds	Last checked	Data type	Link
ArrayExpress^12^	—	—	Raw	ebi.ac.uk/arrayexpress
BindingDB^13^	717,572	04-2019	Curated	bindingdb.org
BioSamples^14^	—	—	Raw	ebi.ac.uk/biosamples
ChEBI^15^	55,660	04-2019	Curated	ebi.ac.uk/chebi
ChEMBL^16^	1,879,206	04-2019	Curated	ebi.ac.uk/chembl
ChemIDPlus^17^	421,602	04-2019	Curated	chem.nlm.nih.gov/chemidplus
ChemSpider^18^	~71 million	04-2019	Curated	chemspider.com
CompTox^19^	~870,000	04-2019	Raw/Curated	comptox.epa.gov/dashboard
CTD^20^	15,913	04-2019	Curated	ctdbase.org
DrugBank^21^	11,926	04-2019	Curated	drugbank.ca
ExpressionAtlas^22^	—	—	Raw/Curated	ebi.ac.uk/gxa/home
GEO^23^	—	—	Raw	ncbi.nlm.nih.gov/geo/
HMDB^24^	114,100	04-2019	Curated	hmdb.ca
HSDB^25^	6,016	04-2019	Curated	toxnet.nlm.nih.gov/cgi-bin/sis/htmlgen?HSDB
PRIDE^26^	—	—	Raw	ebi.ac.uk/pride/archive/
PubChem^1^	>97,400,000	04-2019	Raw/Curated	pubchem.ncbi.nlm.nih.gov
T3DB^27^	3,678	04-2019	Curated	t3db.ca
UniProt^28^	—	—	Curated	uniprot.org
ZINC^29^	>100,000,000	04-2019	Curated	zinc15.docking.org

Abbreviations:

CASRN - Chemical Abstract Service Registry Number;

ChEBI - Chemical Entities of Biological Interest;

FAIR - Findable, Accessible, Interoperable and Reusable;

InChI - International Chemical Identifier;

InChIKey - International Chemical Identifier Key;

IUPAC - International Union of Pure and Applied Chemistry;

SPARQL - SPARQL Protocol and RDF Query Language;

SMILES - Simplified Molecular Input Line Entry System;

RESTFul API - Representational State Transfer Application Programming Interface;

RDF - Resource Description Framework.

## Identifying Data in Compound-Specific Resources

A chemical compound can be referenced with many identifiers, such as a trade name, a generic name, a systematic IUPAC name, a registry number (e.g. CASRN), or a unique database identifier and its structure-derived representations, i.e. structural identifiers: InChI, InChIKey and SMILES. Any of the above can potentially be used to search for a compound within an online resource, but researchers need to be careful about the variability between resources. For example, the compound *rosiglitazone* has 157 depositor-supplied synonyms in PubChem, but only two synonyms in ChEBI. Predictably, the PubChem depositor-supplied synonym for *rosiglitazone* termed *Gaudil* failed to recognize the compound in ChEBI.

Structural identifiers, intuitively, should be the most unique identifiers of a compound, but disparity between the resources still exists. Among eleven resources that reported SMILES (BindingDB, ChEBI, ChEMBL, ChemIDPlus, ChemSpider, CompTox, CTD, DrugBank, HMDB, HSDB, PubChem, T3DB and ZINC15), we found 8 different SMILES for *rosiglitazone* and *tamoxifen*, 5 for *aspirin* and 3 for *valproic acid*. A single InChIKeys was observed for *aspirin*, *valproic acid* and *tamoxifen* but three different ones for *rosiglitazone*. IUPAC systematic names were only reported in ChEBI, ChemSpider, CompTox, DrugBank, HMDB, PubChem and T3DB and demonstrated the largest variability: 3 different names for *aspirin*, 4 for *rosiglitazone*, 1 for *valproic acid* and 5 for *tamoxifen*. UniChem^4^ provides a cross-referencing service connecting 39 individual database identifiers of various resources using InChIKeys but this service is only useful when one already knows the compound’s database identifier or the InChIKey. Currently, it cannot be used with other structural identifiers or compound names.

InChIKey was the most unique identifier among the various databases, possibly because InChI is derived from a single algorithm, whereas several proprietary and open-source algorithms exist for SMILES, whose implementations differ from one another^5^. Although widely used, we did not look at CASRN because the accuracy of CASRN in the public domain is not absolute and reliable information can only be accessed by paid services provided by the Chemical Abstract Service (CAS)^6^.

## Identification of Compound Data in Omics Databases

The identity of chemical compounds reported in omics experiments can be ambiguous since compounds are often mentioned by name without the accompanying structure representations^3^. We investigated this issue by searching a series of omics data resources using structural identifiers of the compounds in Table 1 as reported in ChEBI, using web-based free-text searches (ArrayExpress, ExpressionAtlas, BioSamples, GEO and PRIDE). We were able to retrieve data for all model compounds from at least four resources using compound names. In addition, the IUPAC systematic names of *aspirin*, *rosiglitazone* and *valproic acid* retrieved datasets from ArrayExpress, BioSamples and GEO. Interestingly, in BioSamples, we were able to retrieve datasets *for valproic acid* also with SMILES. These datasets, however, actually corresponded to the sodium salt of *valproic acid*, which has a slightly different SMILES representation in ChEBI compared to *valproic acid*. Confusingly, these samples were not retrieved when the compound name was used instead.

This highlights that, at present, the best way to identify compound-related data from omics resources is with compound names, which requires researchers to exhaust all compound synonyms. To understand this variability between annotations in sample labels, we retrieved the name, synonyms and structural identifiers for each of our model compounds from the ChEMBL public SPARQL endpoint. These were used to identify samples and labels in the BioSamples database through its public SPARQL endpoint. For *rosiglitazone* and *tamoxifen*, only the samples with the respective name was found in any of the sample labels. For aspirin, samples were found using *aspirin*, *asparin*, *asprin*, *levius* and *measurin*. Surprisingly, the compound name *acetylsalicylic acid* was not found in any of the sample labels. *Valproic acid* retrieved results also for *valproate*, *depakote* and *44089*. The latter is a synonym of *valproic acid* in ChEMBL but none of the associated samples were actually associated to *valproic acid*. Of note, all the samples retrieved were unique, i.e. alternative compound labels were not used to annotate the same sample.

## Identification of *in vitro* Compound Data

One approach to identify *in vitro* data in public resources is to browse the study descriptions for references of *in vitro* experiment related keywords like “*in vitro”*, “cell-line” or specific cell-line names (e.g. “HeLa”). ChEMBL provides a web-based search, which allows one to retrieve data on compounds associated with specific cell-lines or *in vitro* assays. Because this approach is not scalable, most public resources also provide access through bulk data downloads, or programmatically through RESTful API or RDF technologies.

In a RESTful query, the data request is constructed into a single URL which is simple to use and platform independent. Out of the 19 resources in our study, 10 provided free access to their RESTful API. The DrugBank API can be accessed for a fee. Data in RDF compatible formats can be supplied as a bulk download, or through public SPARQL endpoints, which facilitate querying the service provider directly, thus always retrieving the most up-to-date data. In our study, only BioSamples, ChEMBL, ExpressionAtlas and UniProt provided a public SPARQL endpoint. Acquiring data using a SPARQL endpoint can be slower compared to RESTful data access, since the latter is better optimized for specific, recurrent query requests. In contrast, SPARQL queries have the benefit of being customizable, providing flexibility that caters to the researchers’ unique needs. Also, since RDF is an inherent part of the “linked data” concept, it can be used to find relationships between datasets in different resources. This is useful for data integration purposes, such as connecting a compound’s effect in one resource to its physicochemical properties in another.

Ontology terms can be used to directly associate and retrieve samples with keywords related to *in vitro* experiments. Using BioSamples’ public SPARQL endpoint as our target database, we found samples for all our model compounds using ChEBI universal reference identifiers (URI) (Table 1). We were also able to find data for our sample compounds retrieved with ChEBI ontology terms, that had been annotated with the molarity unit term (http://purl.obolibrary.org/obo/UO_0000061, Units of measurement ontology, UO^7^) and the cell-line ontology term (http://www.ebi.ac.uk/efo/EFO_0000322, Experimental Factor Ontology, EFO^8^), both indicators of *in vitro* assays. Using the latter, we were able to identify several examples of *rosiglitazone* and *tamoxifen* samples and a single example for *valproic acid*. With the exception of these few examples, we observed that most data for our compounds had been deposited without associated ontology terms. Nevertheless, we are confident that further uptake of the ontologies and improved annotations will be a powerful feature in future search strategies leading to increased data integration capabilities.

## Final Thoughts

**Fig. 1:**
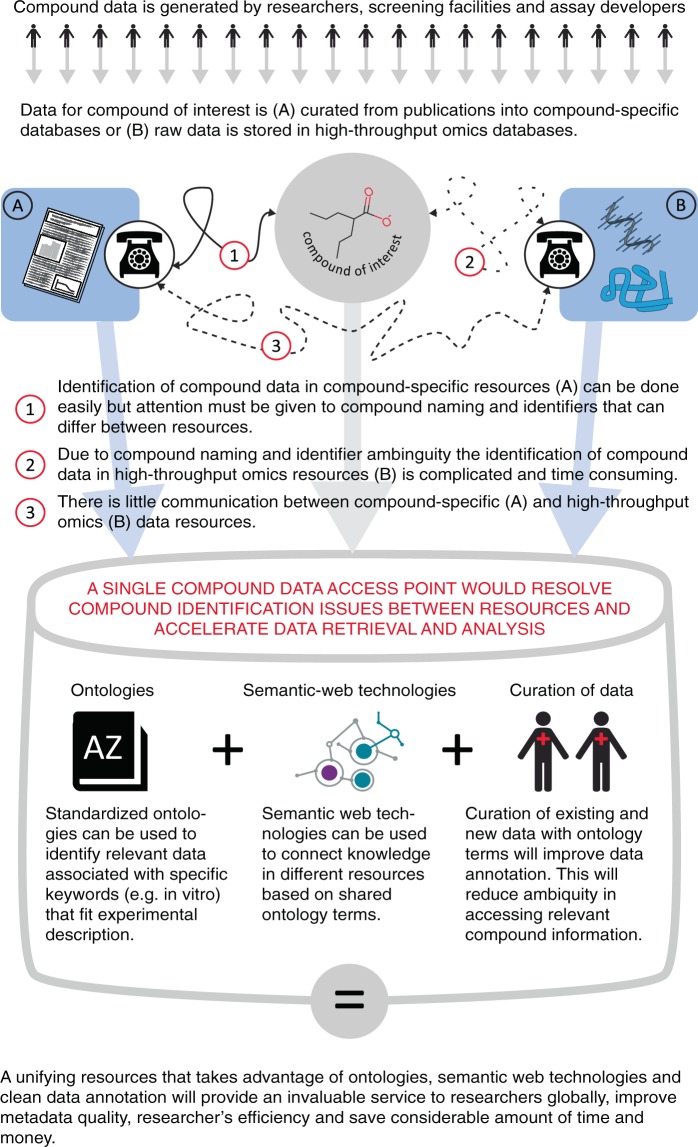
A graphics illustrating the problems of integrating knowledge between compound of interest and different types of data resources. The problems can be solved with integrated approaches using ontologies, semantic-web technologies and better annotation of the data.

There already exists a substantial corpus of resources that contain data on a large number of chemical compounds. These data and their sources are diverse and they need to be integrated in order to attain a complete understanding on a compound (Fig. 1). Accessing published data with correct compound information is essential. The problems encountered in accessing data on our model compounds, demonstrate, that using the results from publications stored in public resources and cross-referencing them with omics data still requires substantial investigative capacity. Efforts similar to SourceData^9^, that allows to annotate already published figures in existing publication, and RepositiveIO (https://repositive.io/), that makes improving metadata a crowd-sourced task, could provide a potential remedy. Would the efforts necessary for general accession to *in vitro* compound data be worth the money and time? Considering the success of UniProt which incorporates extensively curated and trustworthy protein data, the answer is yes. Indeed an analysis published in the EMBL-EBI value report^10^ estimated 46% increase in research efficiency for scientists accessing information relevant to their research question. With around 400,000 unique visitors per month, the reported estimation had an enormous cost-effect benefit for the researcher community. The interest in chemical compounds is even bigger: PubChem alone receives about 1 million unique users per month^11^. This highlights the need for an improved resource that would enhance the efficiency and speed of accessing raw and analyzed compound data in a reliable, simplified and intuitive manner. It would allow researchers to focus on data analysis and its interpretation instead of collection and curation.
